# Polypharmacy in chronic diseases–Reduction of Inappropriate Medication and Adverse drug events in older populations by electronic Decision Support (PRIMA-eDS): study protocol for a randomized controlled trial

**DOI:** 10.1186/s13063-016-1177-8

**Published:** 2016-01-29

**Authors:** Andreas Sönnichsen, Ulrike S. Trampisch, Anja Rieckert, Giuliano Piccoliori, Anna Vögele, Maria Flamm, Tim Johansson, Aneez Esmail, David Reeves, Christin Löffler, Jennifer Höck, Renate Klaassen-Mielke, Hans Joachim Trampisch, Ilkka Kunnamo

**Affiliations:** 1 Institute of General Practice and Family Medicine, Witten/Herdecke University, Alfred-Herrhausen-Str. 50, 58448 Witten, Germany; 2 South Tyrolian Academy of General Practice, Bolzano, Italy; 3 Institute of General Practice, Family Medicine and Preventive Medicine, Paracelsus Medical University, Salzburg, Austria; 4 Centre of Primary Care, University of Manchester, Manchester, UK; 5 Institute of General Practice, Rostock University Medical Centre, Rostock, Germany; 6 Department of Medical Informatics, Biometry and Epidemiology, Ruhr University, Bochum, Germany; 7 Duodecim Medical Publications, Helsinki, Finland

**Keywords:** Polypharmacy, Electronic decision support, Primary care, Multimorbidity, Inappropriate prescribing, Randomized controlled trial

## Abstract

**Background:**

Multimorbidity is increasing in aging populations with a corresponding increase in polypharmacy as well as inappropriate prescribing. Depending on definitions, 25-50 % of patients aged 75 years or older are exposed to at least five drugs. Evidence is increasing that polypharmacy, even when guidelines advise the prescribing of each drug individually, can potentially cause more harm than benefit to older patients, due to factors such as drug-drug and drug-disease interactions. Several approaches reducing polypharmacy and inappropriate prescribing have been proposed, but evidence showing a benefit of these measures regarding clinically relevant endpoints is scarce. There is an urgent need to implement more effective strategies. We therefore set out to develop an evidence-based electronic decision support (eDS) tool to aid physicians in reducing inappropriate prescribing and test its effectiveness in a large-scale cluster-randomized controlled trial.

**Methods:**

The “**P**olypharmacy in chronic diseases–**R**eduction of **I**nappropriate **M**edication and **A**dverse drug events in older populations” (PRIMA)-eDS tool is a tool comprising an indication check and recommendations for the reduction of polypharmacy and inappropriate prescribing based on systematic reviews and guidelines, the European list of inappropriate medications for older people, the SFINX-database of interactions, the PHARAO-database on adverse effects, and the RENBASE-database on renal dosing. The tool will be evaluated in a cluster-randomized controlled trial involving 325 general practitioners (GPs) and around 3500 patients across five study centres in the United Kingdom, Germany, Austria and Italy. GP practices will be asked to recruit 11 patients aged 75 years or older who are taking at least eight medications and will be cluster-randomized after completion of patient recruitment. Intervention GPs will have access to the PRIMA-eDS tool, while control GPs will treat their patients according to current guidelines (usual care) without access to the PRIMA-eDS tool. After an observation time of 2 years, intervention and control groups will be compared regarding the primary composite endpoint of first non-elective hospitalization or death.

**Discussion:**

The principal hypothesis is that reduction of polypharmacy and inappropriate prescribing can improve the clinical composite outcome of hospitalization or death. A positive result of the trial will contribute substantially to the improvement of care in multimorbidity. The trial is necessary to investigate not only whether the reduction of polypharmacy improves outcome, but also whether GPs and patients are willing to follow the recommendations of the PRIMA-eDS tool.

**Trial registration:**

This trial has been registered with Current Controlled Trials Ltd. on 31 July 2014 (ISRCTN10137559).

**Electronic supplementary material:**

The online version of this article (doi:10.1186/s13063-016-1177-8) contains supplementary material, which is available to authorized users.

## Background

The prevalence of multimorbidity is substantial and affects more than half of the population aged 75 years or older in developed countries [1]. Cardiovascular disease (including coronary heart disease, cerebrovascular disease and peripheral vascular disease), heart failure, hypertension, atrial fibrillation, diabetes mellitus type 2, musculoskeletal pain, chronic obstructive pulmonary disease (COPD), and mental diseases like depression and dementia most often co-occur, with heart failure showing the highest rate of comorbidity, being accompanied by an average of 2.9 additional chronic diseases [2]. It is generally recommended to treat each chronic condition in accordance with disease-specific guidelines. However, most clinical practice guidelines do not modify or discuss the applicability of their recommendations for older patients with multiple diseases and following all guidelines for each and every drug a patient is taking will inevitably lead to polypharmacy [3]. Even though the term “polypharmacy” is frequently used to describe the use of multiple medications, a clear definition is still lacking. The variety of definitions reflects the difficulty in setting an accurate cut-off point. According to the medical literature review of Bushardt et al., the most common cut-off point is the use of more than five medications [4]. Depending on definitions and setting, between 25 and 50 % of all patients age 75 years or older are exposed to five or more drugs [5–8]. The clinical trial evidence of benefit for most drugs is based on studies of mostly younger, fitter subjects with a life expectancy of many years, even though these drugs are most often prescribed for older patients with multimorbidity, and the findings of these studies may not be so applicable due to drug-drug and drug-disease interactions as well as aging processes and a reduced life expectancy [9, 10].

Polypharmacy is associated with an increased risk for medication errors [11] and adverse drug events (ADE) [12, 13] which in turn are frequent causes of hospitalization [14]. Various international studies report a hospitalization rate due to ADE of between 2.4 and 16.6 %, depending largely on the age group evaluated [15–17]. Polypharmacy and inappropriate medication have been shown to contribute substantially to the burden of morbidity, hospitalization and death [18]. Up to 50 % of ADE and ADE-related hospitalizations are judged to be preventable by avoiding inappropriate prescribing [19].

Although exact figures are not available for all countries, it may be assumed that between 60 and 80 % of all patient prescriptions are initiated by general practitioners (GPs). In Germany, 67.6 % of all prescriptions covered by statutory public health insurance are attributable to GPs [20]. Any intervention aimed at the reduction of polypharmacy will, therefore, be most effective if targeted at GPs.

A number of diverse approaches to reduce polypharmacy and inappropriate prescribing have been proposed, but no convincing evidence from sufficiently powered randomized controlled trials exists regarding their practicality for use in primary care or their impact on clinically relevant endpoints. There is limited evidence that the Beers Criteria list of potentially inappropriate medications (PIM) for older patients has some associations with adverse outcomes, but this evidence is mostly based on retrospective cohort studies, and there is no study showing that avoiding drugs on a PIM list for older patients like the Beers list will improve outcomes [21]. There is also preliminary evidence that the prudent reduction of polypharmacy using the Garfinkel algorithm may be beneficial to patients, but Garfinkel used a non-randomized design in his studies [22, 23]. Similarly, it has been shown that the application of STOPP-criteria is associated with ADE [24, 25] and a reduction of PIMs [26], but a randomized controlled trial proving that this also improves clinical outcome does not exist.

Use of a simple interdisciplinary medication review has been shown to lead to the reduction of inappropriate prescribing and costs, but there was no effect on clinically relevant patient outcomes, possibly due to a lack of power and insufficient observation time [27].

Some authors suggest a “process of deprescribing,” prioritizing drugs in a patient with polypharmacy according to each drug’s risk/benefit ratio, but likewise we do not have evidence from randomized trials supporting this strategy [28].

A recently updated Cochrane review concluded that both the application of the “medication appropriateness index” (MAI) and the Beers list appeared to improve medication appropriateness, but effects on outcome were inconsistent, and the quality of evidence has been graded as low or very low [29]. Moreover, most of the studies included in the review were carried out in a hospital or nursing home setting.

There is a strong need for improved interventions to reduce inappropriate prescribing and polypharmacy, especially in the primary care setting. We therefore set out to develop an electronic decision support (eDS) tool based on current best evidence and targeted at the primary care setting to help GPs identify and reduce inappropriate prescribing and polypharmacy in older patients. The principal intervention will be the recommendation for drug discontinuation or modification by the “Polypharmacy in chronic diseases–Reduction of Inappropriate Medication and Adverse drug events in older populations” (PRIMA)-eDS tool (see Methods section for further description of the tool).

### Objectives of the study

Our principal hypothesis is that the reduction of polypharmacy and inappropriate prescribing will improve clinical outcomes for older patients. The primary objective is to test this hypothesis by the evaluation of the PRIMA-eDS tool in a multicenter cluster-randomized controlled trial with the combined primary endpoint of first non-elective hospital admission or death (see Table 1).

**Table 1:** PICO research question of the PRIMA-eDS trial

PICO-Item	Research question
P (patients)	Patients aged ≥75 years taking ≥8 drugs in primary care
I (intervention)	GPs of enrolled patients with access to the PRIMA-eDS tool
C (comparison)	GPs of enrolled patients without access to the PRIMA-eDS tool (usual care)
O (outcome)	Non-elective hospitalization or death within an observation time of 2 years

*PRIMA-eDS* Polypharmacy in chronic diseases–Reduction of Inappropriate Medication and Adverse drug events in older populations by electronic Decision Support

Secondary objectives are to evaluate the effectiveness of the decision support tool regarding all-cause mortality and non-elective hospital admission as single endpoints as well as a number of secondary outcomes (see Methods section).

## Methods/Design

### Trial design

The PRIMA-eDS trial is a multicentre cluster-randomized controlled trial with an observation time of 2 years using GP practices as clusters of randomization. It will be conducted at five study centres recruiting GP practices in their corresponding region/country. The principal trial centre and coordinator of the study is the Institute of General Practice and Family Medicine of Witten/Herdecke University (Germany). The other four study centres are the University of Manchester (United Kingdom), the Rostock University Medical Centre (Germany), the Paracelsus Medical University (Austria), and the South Tyrolean Academy of General Practice (Italy).

### Ethics approval

The study has been approved by the five local ethics committees: 1. Ethikkomission der Universität Witten/Herdecke, 3 December 2013, ref. 103/2013; 2. NRES Committee North West – Greater Manchester East, 6 June 2014, ref. 14/NW/0197; 3. Ethikkommission für das Bundesland Salzburg, 15 September 2013, ref. 08.04.2014 (415-E/1509/20-2014); 4. Ethikkommission der Universitätsmedizin Rostock, 3 February 2014, ref. A 2014-0020; and 5. Comitato etico di Belluno (Azienda ULSS), 19 June 2013, ref. 305388-2.

### Setting

The PRIMA-eDS trial will be located in the primary care setting including community as well as nursing home resident older patients taking at least eight drugs (active ingredients with a systemic effect, combination drugs are counted according to the number of active ingredients). We chose the inclusion-criterion of at least eight drugs because the sample size calculation of this trial is based on the study by Garfinkel where a corresponding number of drugs was taken by the participants [23]. Table 2 provides a list of the five study centres and their corresponding settings for recruitment of GP practices.

**Table 2:** Study centres and settings of the trial

Study centre	Settings for the recruitment of GP practices
University of Witten/Herdecke	Network of teaching and research practices of the university; list of all GP practices providing care for patients with statutory public health insurance in the region of South Eastern North-Rhine-Westfalia
Rostock University Medical Centre	List of all GP practices providing care for patients with statutory public health insurance in the region of Mecklenburg-Western Pomerania and parts of Berlin
Paracelsus Medical University	List of all GP practices of the province of Salzburg; list of all GP practices providing care for patients with statutory public health insurance in the region of South Eastern Bavaria
South Tyrolian Academy of General Practice	Research network of primary care practices of the Scuola Veneta di Medicina Generale (SVeMG)
University of Manchester	Primary Care Research Network East of England (PCRN EoE) and others

### Recruitment of GP practices and patients

Each study center will inform all GPs of a selected region or a GP practice network (see Table 2 for details) about the trial, first in writing, and then followed by a telephone call, and invite them to participate in the study. After giving informed consent, GPs are encouraged to identify all patients of their GP practice eligible to participate from the electronic health record of the GP practice and check these for inclusion and exclusion criteria.

### Inclusion criteria

- Age 75 years or older
- Intake of at least eight drugs including drugs prescribed by the GP or any other physician (e.g., specialist) and non-prescription drugs (including PRN (pro re nata) medication)

### Exclusion criteria

Patients will be excluded if their life expectancy is assumed to be less than 1 year (assumption based on GP judgment). Other exclusion criteria are ongoing chemotherapy and/or ongoing therapeutic radiation for systemic malignant disease, and dementia with inability to provide informed consent. All or a sample of the eligible patients at the discretion of the GP are then invited in writing or by personal contact to participate in the trial. After giving informed consent in accordance with the declaration of Helsinki, patients are continuously included in the study. GPs are instructed to aim for a minimum of eight and a maximum of 15 patients to keep cluster size constant. The flow of recruitment is depicted in Additional file 1: Figure S1.

### Randomization

The unit of randomization will be the GP practice, such that each participating GP and their patients will be randomized to either have use of the PRIMA-eDS tool (intervention), or not (control). This avoids any risk of contamination that would occur if GPs were using the tool with some of their patients but not others. The intended GP practice cluster size is around 11 patients (see sample size calculation). To assure concealment of allocation, each GP practice reports to the Department of Medical Informatics, Biometry and Epidemiology, Ruhr University, Bochum, Germany as soon as patient recruitment has been completed, and is then cluster-randomized by computerized sequence generation. Thus, neither patients nor GPs will know whether they will take part as an intervention or control cluster at the time of recruitment. To avoid over-representation of a single study center in the intervention group, randomization will be stratified by study center.

### Blinding

Inherent to the study protocol GPs and patients cannot be blinded. The analyst will be kept blind to GP practice allocation.

### Intervention

The principal intervention will be the recommendation for drug discontinuation or modification by the PRIMA-eDS tool. This is a novel decision support tool which has been developed by the PRIMA-eDS consortium to make current best evidence regarding drug treatment of chronic diseases in older patients available to GPs in daily practice. The tool analyses the patientʼs diagnoses, current medication, symptoms, biometric measurements (e.g., body mass index, blood pressure) and laboratory values, performs an electronic comprehensive medication review, and returns recommendations for drug discontinuation or modification based on the following components:

- Indication and contraindication check for all prescribed drugs according to the diagnoses provided and the officially approved indications by the European Medicines Agency (EMA)
- Forty rules and recommendations based on systematic reviews of clinical trial and observational study evidence on usage of the most commonly prescribed drugs in older patients [30]
- Ninety-five rules and recommendations taken from the Evidence-Based Medicine electronic Decision Support (EBMeDS) database (http://www.ebmeds.org/web/guest/home). These recommendations in turn are derived from the NICE-approved Evidence Based Medicine Guidelines (EBMG; http://www.duodecim.fi/web/duodecimpublications/ebmg) to assure regular updating
- RENBASE renal dosing decision support software (http://www.medbase.fi/en/professionals/renbase)
- Swedish, Finnish, INteraction X-referencing (SFINX) interaction check decision support software (www.medbase.fi/en/professionals/sfinx) [31]
- PHARAO decision support software on adverse drug reactions (http://www.medbase.fi/en/professionals/pharao)
- Check of drugs listed in the European list of potentially inappropriate medication for older patients [32]

The PRIMA-eDS tool supports GPs to reconsider, modify or stop the use of certain drugs in older patients with polypharmacy. Recommendations provided by the tool do not replace careful clinical consideration by physicians, nor clinical guidelines, but aim at supporting clinical decisions.

After entering diagnoses, current medication including drugs that have not been prescribed by the GP, and other patient data (current symptoms, biometric measurements and laboratory values), the GP will receive an on-screen “Comprehensive Medication Review” report, generated by the PRIMA-eDS tool and detailing all the relevant prescribing recommendations. The patient’s list of medications can then be reviewed and revised accordingly by the GP in a shared decision-making process with the patient. A record will be made of which recommendations were adopted. GPs will be explicitly instructed that any decision to continue or discontinue a drug according to or against the advice of the PRIMA-eDS tool remains at the discretion of the GP and the patient. All intervention GPs will be trained to use the PRIMA-eDS tool. Training options will include personal training, group training, webinars and a handbook.

Before the start of the trial, the tool has been evaluated in a feasibility study which has been presented at the 16^th^ annual meeting of the German Network for Evidence-based Medicine [33]. The tool as a whole has not yet been tested for validity or reliability, but substantial work has demonstrated the feasibility and applicability of the various components of the tool. The use of clinical decision support (CDS) for promoting appropriate, safe, and cost-effective drug use has become common as an inbuilt feature of advanced electronic health record (EHR) systems [34]. CDS can also be implemented as an externally offered service that is integrated with an EHR [35]. The EBMeDS CDS system (www.ebmeds.org) is a service developed by Duodecim Medical Publications Ltd. since 2003. An early version of EBMeDS containing a variety of decision support rules was evaluated in a randomized controlled trial [36]. Databases on drug-drug interactions and renal dosing developed by Medbase Ltd. have been included in EBMeDS. The design and usability of these databases have been described [31, 37, 38], and their impact evaluated [39, 40]. Because of its modular design, comprehensiveness and easy integration with EHRs and case-report forms, EBMeDS was selected as the platform for developing the PRIMA-eDS tool. All components of the tool are regularly updated during the trial by the corresponding provider and by the PRIMA-eDS consortium.

### Control

GPs in the control group will be asked to record the medication and other data of the participating patients at the same time points as the intervention group. Control group GPs will provide their patients with the care they would normally receive (usual care), including adhering to any relevant current guidelines, some of which may be country-specific, but without the help or recommendations from the PRIMA-eDS tool which will not be available to them.

### Documentation and monitoring

#### Baseline-examination

Shortly after consent is obtained, the GPs will assess the following parameters and record all data in each patient’s electronic case report form (eCRF):

- Age, sex, height, weight, systolic and diastolic blood pressure
- All drugs with a systemic effect
- All diagnoses
- Current symptoms (within last month)
- Falls within the last 3 months requiring medical attention
- Creatinine, blood glucose, alanine aminotransferase (ALT), hemoglobin A1c (HbA1c), platelet count, international normalized ratio (INR), cholesterol, low-density lipoprotein (LDL)-cholesterol, high-density lipoprotein (HDL)-cholesterol, triglycerides, potassium, sodium. With the exception of creatinine level, GPs should not initiate additional laboratory examinations solely for the study due to ethical reasons and to avoid contamination in this pragmatic trial. They should rather enter the latest values available in the health record and include the date of the measurement. If the tool requires a laboratory value for a recommendation, it provides this information to the GP. The GP can then decide whether to perform the laboratory examination or not
- Smoking behavior
- Medical procedures (e.g., stenting, heart valve replacement)
- Frailty according to the Clinical Frailty Scale [41]
- Quality of life questionnaire (SF-12v2) to be filled out by the patient [42]. This will be administered by the GP to the patient on paper. The filled out forms will then be sent to the study centre for electronic data entry

### Follow-up

At follow-up visits (at 8 and 16 months) and final examination (24 months) the same data will be recorded again for all patients. One exception is the quality of life questionnaire (SF-12v2), which will be collected at 8 and 24 months only. GPs in the intervention group will conduct further medication reviews using the PRIMA-eDS tool for their participating patients, and make the medication changes they think are appropriate, at each follow-up and final visit. Control group GPs will continue to provide usual care to their patients.

### Monitoring

Monitoring is performed according to a prespecified monitoring charter. Independent monitors selected by the corresponding study centers will conduct all monitoring. Inclusion and exclusion criteria will be checked electronically. The monitors will control whether the informed consent forms of all participants are correctly filled out and signed. All other data will be monitored at each follow-up visit in a random sample of 10 % of the GPs. On-site visits are performed at T0 and T3. Monitors will compare the entries of all study patient records to the entries in the eCRF regarding age, sex and medication. In a random sample of three patients, monitors will check the eCRF entries of the medication plus doses, diagnoses, falls, and laboratory results as well as height and weight, and compare these data to the patient record. Additionally at T3, monitors will check all reported and not reported non-elective hospital admissions as well as adherence to protocol visits with the practice record. At T1 and T2 monitors will check this via phone verification. Furthermore, at T1 to T3, monitors will monitor 100 % of the reported dropouts via phone verification, and in a random sample of 10 % of the practices they will check the reported hospital admissions and deaths of all patients of the practice.

For safety purposes, the PRIMA-eDS consortium will appoint a Safety and Data Monitoring Committee (SDMC) consisting of three experienced researchers, including one clinical trial statistician. The committee is independent from the investigators and will review the accumulated trial data. They will perform interim analyses regarding the primary endpoints 4 months after the follow-up visits exclusively for safety reasons and without disclosure of the randomization. Additionally, ADEs will be monitored regularly. The results will be available only to the SDMC. The SDMC will report any recommendation on necessary trial modifications or a trial abort to the principal investigator and the coordinating monitors. The SDMC will recommend termination of the trial if clinically relevant safety concerns arise in either the control or the intervention group. No explicit stopping rules have been developed.

### Primary outcome

The primary outcome is a composite endpoint of first non-elective hospital admission or death during the observation period measured as a binary outcome. This avoids issues of a potential trade-off between these two forms of event and also allows all patients to be included in the primary analysis. Primary outcome measures are recorded by the GPs when they occur or at follow-up visits every 8 months.

### Secondary outcome

Secondary endpoints are all cause mortality, non-elective hospital admission (number of episodes and duration), falls (number and severity), fractures, quality of life (SF-12v2), the number and types of drugs (total number, number discontinued, number not discontinued despite the recommendation to discontinue, number re-administered for symptom control), adverse event rate, and medication costs over the observation period.

### Potential harms and patient safety

We do not expect any specific complications or harms for patients because only non-evidence-based medications will be discontinued. However, there is a slight possibility that the discontinuation of a drug might lead to reappearance or development of new symptoms. In this case, the medication can be restarted at any time at the discretion of the GP. The PRIMA-eDS tool only provides recommendations based on current best evidence. The decision about the medication always remains with the GP in agreement with the patient. Nevertheless, we monitor potential harms. GPs are required to report serious adverse events in the eCRF as soon as they get notice of the event, and all adverse events at the scheduled study visits. They are asked to judge whether the adverse event is associated with the intervention. All serious adverse events will be reported to the SDMC for final analysis and judgment.

### Data management and sample size calculation

Sample size calculation is based on binary analysis of the primary endpoint (non-elective hospital admission or death within 2 years). The study sets out to prove superiority regarding the primary endpoint. Garfinkel observed an 18 % absolute risk reduction achieved by prudent reduction of polypharmacy in a non-randomized controlled trial (from 30 % in the control group to 12 % in the intervention group regarding the annual referral rate to acute care facilities) [23]. This corresponds to a relative risk reduction of 60 % regarding this endpoint.

However, considerably smaller reductions than this still represent substantial clinical and economic benefits; therefore, this study is powered to detect a relative risk reduction of 20 %. We expect to recruit healthier patients than Garfinkel and will use non-elective hospital admission (yes/no) instead of referral rate, because it seems less susceptible to bias. In addition, our primary outcome will combine mortality with hospital admission, since there may be trade-off between these. We have been unable to find empirical data on which to estimate the rate of admission and mortality for our population, but in England in 2010 there was an average of approximately one completed consultant episode for every person in the population aged 75 years or older [43]. The primary outcome of dropouts is coded as an ‘event’ in both groups. Therefore, we consider a low estimate to be half the rate observed by Garfinkel [23]: an annual admission and mortality rate of 15 % of control patients; or 30 % in 2 years. Calculations show that in the event of the actual rate being higher than this, the sample size given below is conservative (ie., larger, rather than smaller, than requirement).

A relative risk reduction of 20 % in the intervention group implies a 2-year event rate of 24 % for that group.

The sample size is calculated according to Hayes et al. [44] for a power of 80 % (1-*β* = 0.8), *α* =5 %, and an assumed intra-cluster correlation coefficient (for similarity in outcomes between patients within GP practices) of no more than 0.01 (derived from an earlier cluster-randomized trial involving GP practices as clusters [45]). Dropouts in both groups will be considered as treatment failures. Further assuming attrition rates of 10 % of patients and 10 % of GP practices over the period of the study, sample sizes required at recruitment are 3542 patients (1771 per arm) across 322 GP practices at a mean of 11 patients per GP practice (Poisson random variation in patient numbers per GP practice is assumed). To operate a practicable number of GP practices and patients per country we are aiming at 325 GP practices (65 per study center) recruiting 3575 patients (11 patients per GP practice). See Additional file 1: Figure S1.

We will record all data in the PRIMA-eDS eCRF and store them in a XML-database in a secure central server provided by Avain technologies (http://www.avaintec.com/?lang=en). To monitor data quality, we will transfer data to the Department of Medical Informatics, Biometry and Epidemiology, Ruhr University, Bochum, Germany, every week throughout the trial. Statistical analyses will only be carried out after the end of the trial, with no interim analysis planned. Upon completion of data collection, Avain technologies will provide a final file for statistical analysis.

### Statistical analysis

Statistical analyses are elaborated in a prespecified statistical analysis plan. The binary primary outcome will be analyzed following intention-to-treat principles; secondary outcomes per protocol. A suitable generalized mixed model will be used to account for cluster randomization. Additionally, the study center as fixed factor is included to avoid confounding through stratified randomization. A two-sided *α* of 5 % will be used throughout.

The intervention could result in mortality rates differing between the two trial arms, which could bias analyses of other outcomes if simple exclusion were to be applied. If we find evidence for differential mortality, analysis of secondary outcomes will employ appropriate adjustment methods [46].

Besides a per protocol analysis of the primary outcome, further sensitivity analyses will be undertaken to assess the stability of the results to the model specification, including time-to-event and counts of events analyses as well as patient and GP practice covariates found to be unbalanced across the two trial arms. Random imputation methods will be used, to investigate the sensitivity of the chosen method for replacement of missing values for dropouts in the primary endpoint.

### Dissemination policy

All results of the trial will be published according to a dissemination plan submitted to the EU with the submission of the proposal. There are no publication restrictions. Authorship eligibility guidelines will be followed. Use of professional writers is not intended. The full protocol, the dataset and the statistical code can be made available upon request.

## Discussion

Current knowledge about optimal drug treatment in older patients with multiple chronic conditions is very limited and the PRIMA-eDS trial can potentially make a substantial contribution in this area. We are confronted with increasing evidence that current prescribing practices, which are highly conducive of polypharmacy in the treatment of multiple chronic diseases, do not achieve the same benefits as are observed in younger people with single diseases and may in fact be quite detrimental to the health of older people with multimorbidity [10]. Very few existing studies have explicitly investigated drug treatments in older patients, and where they have done, have tended to focus on otherwise rather healthy patients. In contrast, the PRIMA-eDS trial will purposely sample and research an older-age population with multiple chronic conditions and many prescribed medicines, and will evaluate whether the polypharmacy and inappropriate prescribing burden can be safety reduced – and health outcomes consequently improved – within a routine primary care context.

Our systematic reviews of the literature on drug benefits and risks (conducted in the context of the development of the PRIMA-eDS tool) will gather the available evidence and evaluate it critically regarding its applicability to an older population with multimorbidity [30]. We envisage that this will kick-start a process of continuous development of new guidelines specifically addressing drug treatments in the context of multimorbidity.

### Limitations of the study

We have explicitly decided to not include any recommendations in the PRIMA-eDS tool for starting patients on additional drugs, and this may be considered a weakness of our study. Our main objective is to demonstrate that the discontinuation of inappropriate drugs can improve patient outcomes. A combined intervention to stop inappropriate and start other (more appropriate) medications would impede us from differentiating out the effect of drug discontinuation.

Furthermore, access to medical records differs by country. Italy and the UK are characterized by a primary care centred health care system, with all medication data and diagnoses available to the GP. In these two countries we expect data from the electronic health records of the GPs to be largely complete. This is not the case in Germany or Austria where patients have direct access to specialists, and not all medication prescribed by a specialist will be known to the GP. We are asking GPs to address medication prescribed by specialists at the study visits to assure completeness of data. Nonetheless, patientsʼ data in these countries carry a risk of being incomplete which might be considered a limitation of our study. However, the problem of incomplete medication data equally affects the intervention and the control group. We therefore believe that due to randomization being stratified by study centre, incomplete medication data will not bias our study results.

### Trial status

Recruitment started in January 2015 and is expected to be completed by 30 September, 2015. The last follow-up visit will be scheduled 2 years after the last patient has been consented into the study.

## Additional file

Additional file 1: Figure S1. Recruitment of 325 practices and 3575 patients for the PRIMA-eDS* trial. Figure describing the flow of recruitment for the PRIMA-eDS trial. (JPG 117 kb)

## Abbreviations

ADE: adverse drug event
ALT: alanine aminotransferase
eCRF: electronic case report form
eDS: electronic decision support
GP: general practitioner
HbA1c: hemoglobin A1c
HDL: high-density lipoprotein
INR: international normalized ratio
LDL: low-density lipoprotein
PIM: potentially inappropriate medication
PRIMA-eDS: Polypharmacy in chronic diseases–Reduction of Inappropriate Medication and Adverse drug events in elderly populations by electronic Decision Support
PRN: pro re nata
SDMC: Safety and Data Monitoring Committee
