# Supplementary figures and images for: Polypharmacy in chronic diseases–Reduction of Inappropriate Medication and Adverse drug events in older populations by electronic Decision Support (PRIMA-eDS): study protocol for a randomized controlled trial
Source: Trials. 2016 Jan 29;17:57. doi: 10.1186/s13063-016-1177-8 (PMC5526277; doi:10.1186/s13063-016-1177-8)

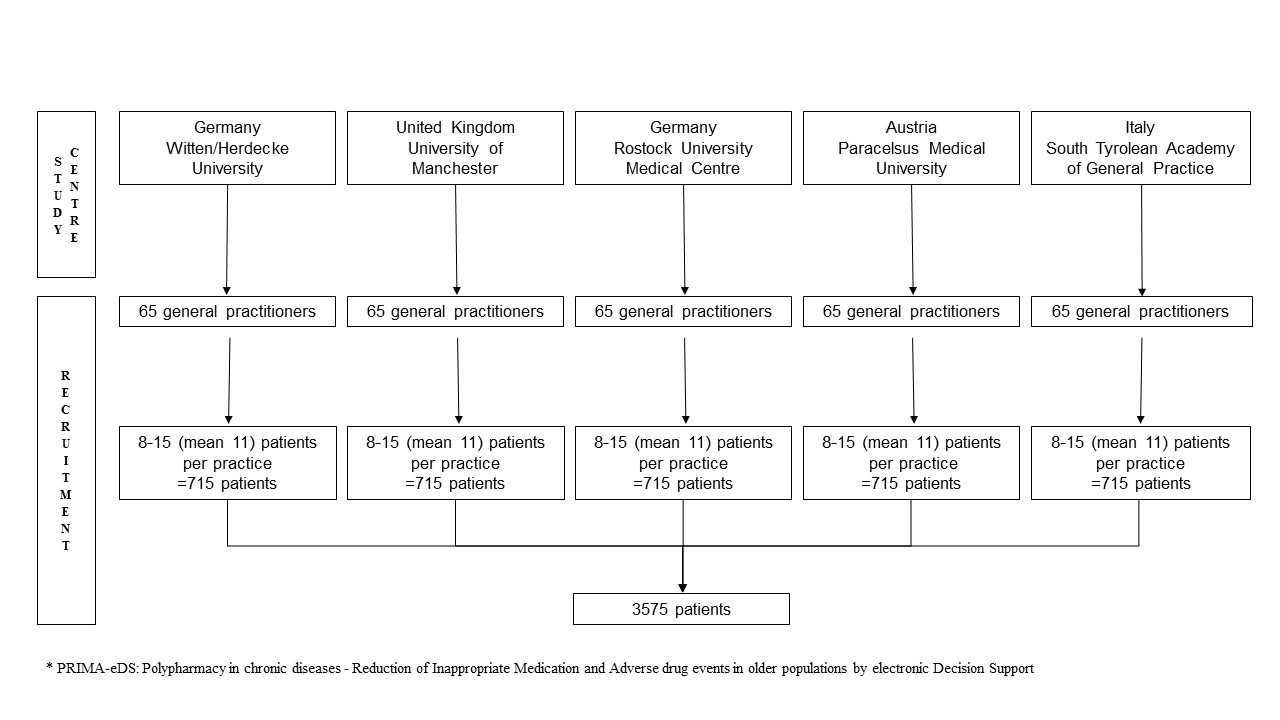

Supplement: Additional file 1: Figure S1. — Recruitment of 325 practices and 3575 patients for the PRIMA-eDS* trial. Figure describing the flow of recruitment for the PRIMA-eDS trial. (JPG 117 kb) [file 13063_2016_1177_MOESM1_ESM.jpg]
